# Supplementary material for: Deletion of wheat alpha-gliadins from chromosome 6D improves gluten strength and reduces immunodominant celiac disease epitopes
Source: Theor Appl Genet. 2025 Apr 8;138(5):94. doi: 10.1007/s00122-025-04882-3 (PMC11978689; doi:10.1007/s00122-025-04882-3)

Supplementary figures

Fig. S1. Model for the separation of polymeric and monomeric prolamins in 50% 1-propanol.

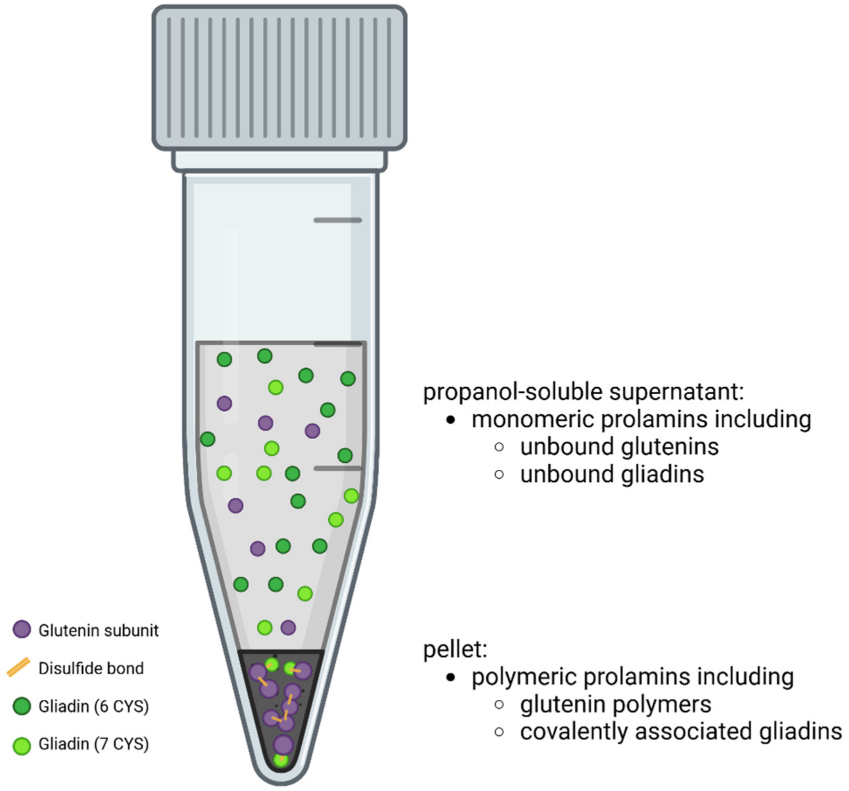

Fig. S2. Haplotype analysis for the α-gliadin locus and flanking regions on chromosome 6A. Tetraploid accessions are indicated in blue. Sequenced genomes are indicated in bold. Data S2.

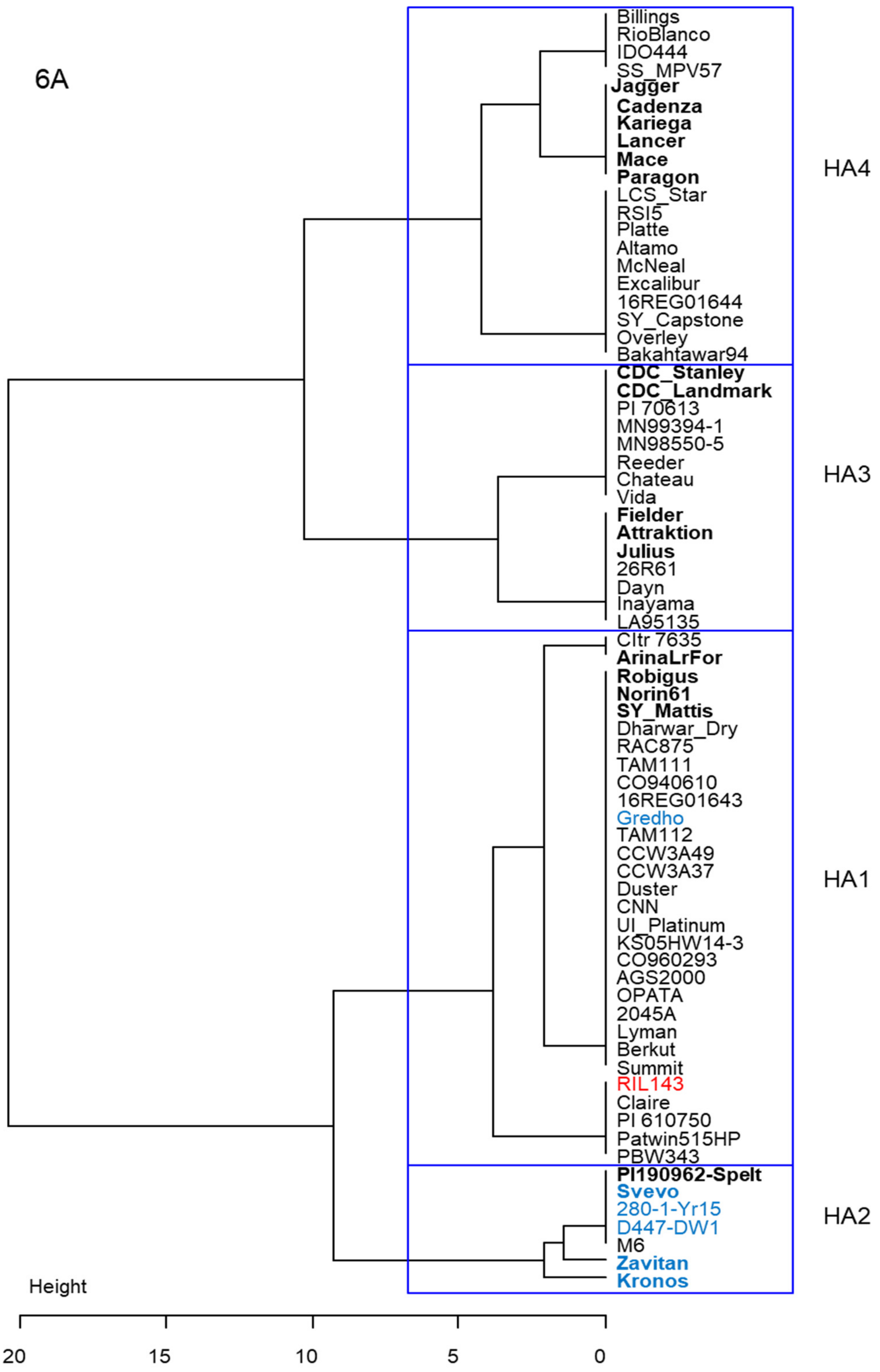

Fig. S3. Haplotype analysis for the α-gliadin locus and flanking regions on chromosome 6B. Tetraploid accessions are indicated in blue. Sequenced genomes are indicated in bold. Data S3.

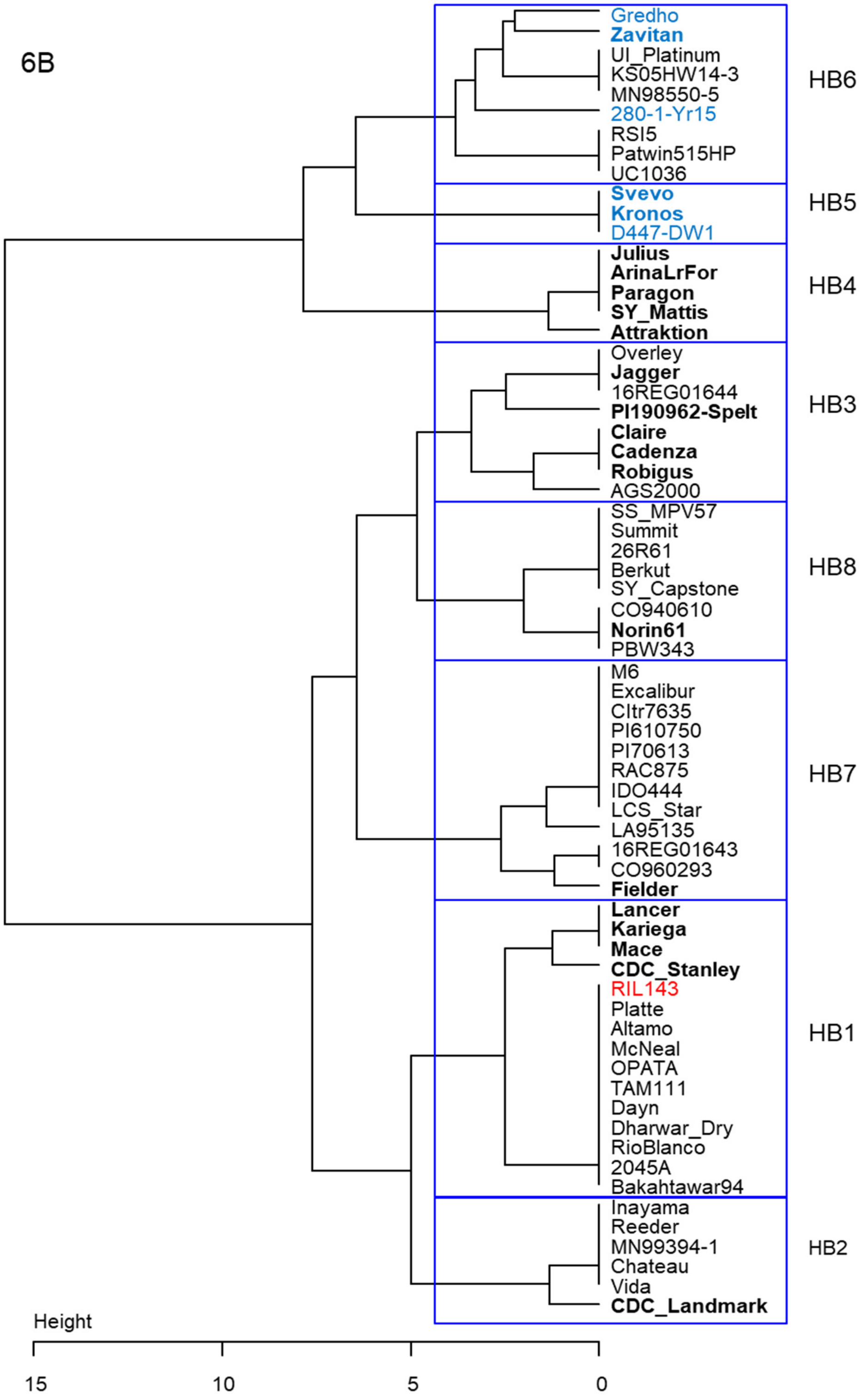

Fig. S4. Haplotype analysis for the α-gliadin locus and flanking regions on chromosome 6D. *Ae tauschii* accessions are indicated in green. Sequenced genomes are indicated in bold. Data S4.

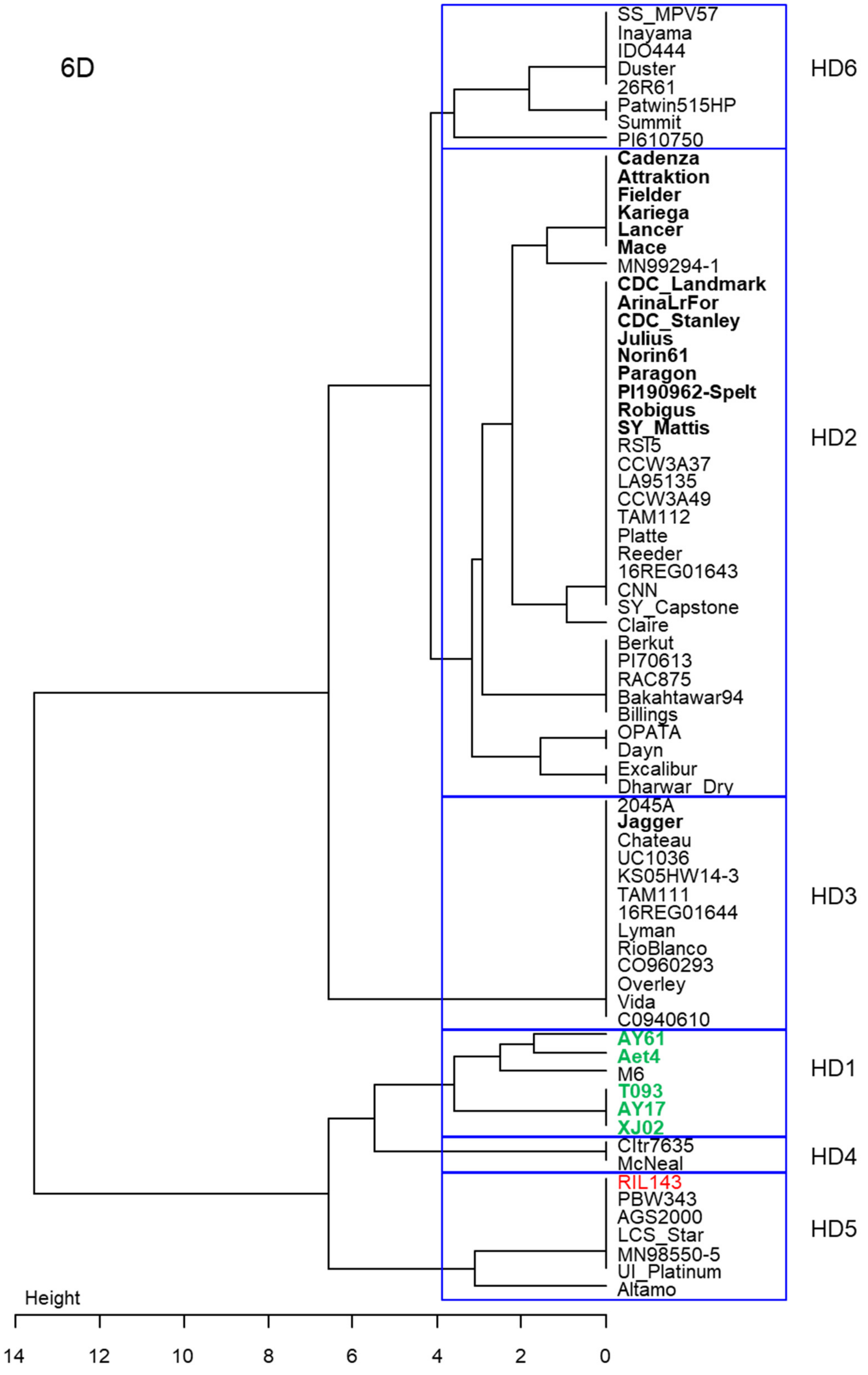

Fig. S5. Wildtype Kronos and *Δgli-B1 Δgli-D1* combined deletion line. The left panel shows normal floral organs in wildtype Kronos and the right panel shows abnormal sterile flowers with no stamens and multiple pistils in the combined *Δgli-B1 Δgli-D1* deletion line.

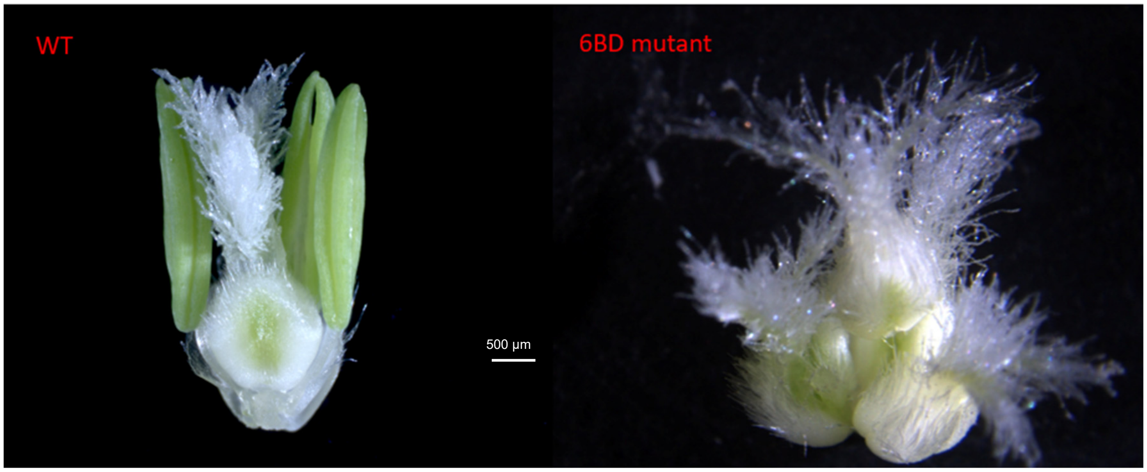

Fig. S6. Extension results. (A) Distance to break (indicates the extensibility of the dough). (B) Force (indicates the strength of the gluten). The control line RIL143 was compared with deletion lines *Δgli-A2, Δgli-B2, Δgli-D2,* and *Δgli-A2 Δgli-D2* in the same genetic background. Means of the deletion lines were compared with control line RIL143 using Dunnett tests. ns= not significant, ** = $P< 0.01$, *** = $P< 0.001$. Error bars (s.e.m.) are based on four blocks. Each genotype within a block was represented by five homozygous sister lines that were averaged and treated as subsamples. Average values of the five subsamples are presented in Table S10.

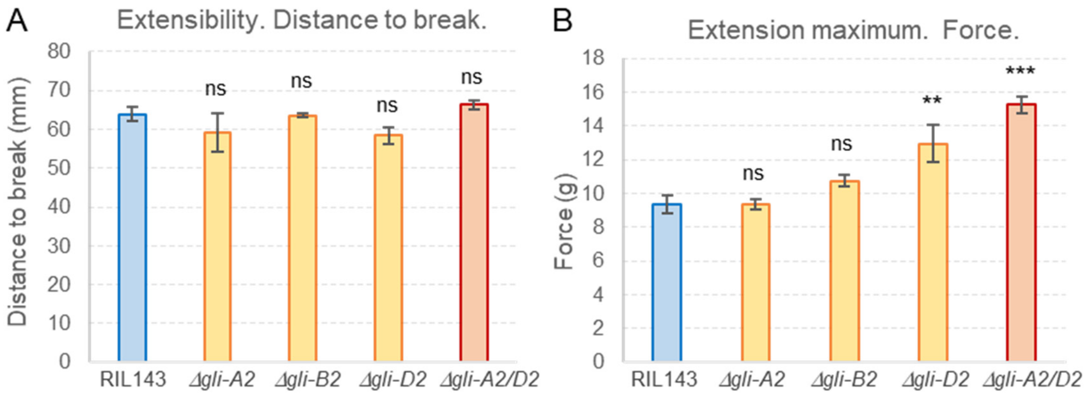

Fig. S7. Alignment between 7-CYS and 6-CYS α-gliadins from Chinese Spring detected in the proteomics study. Genes indicated as CSU were assigned to chromosome 6D based on high similarity to colinear genes in Fielder. Amino acids that separate the 7-CYS from the rest are marked in red and highlighted in yellow.

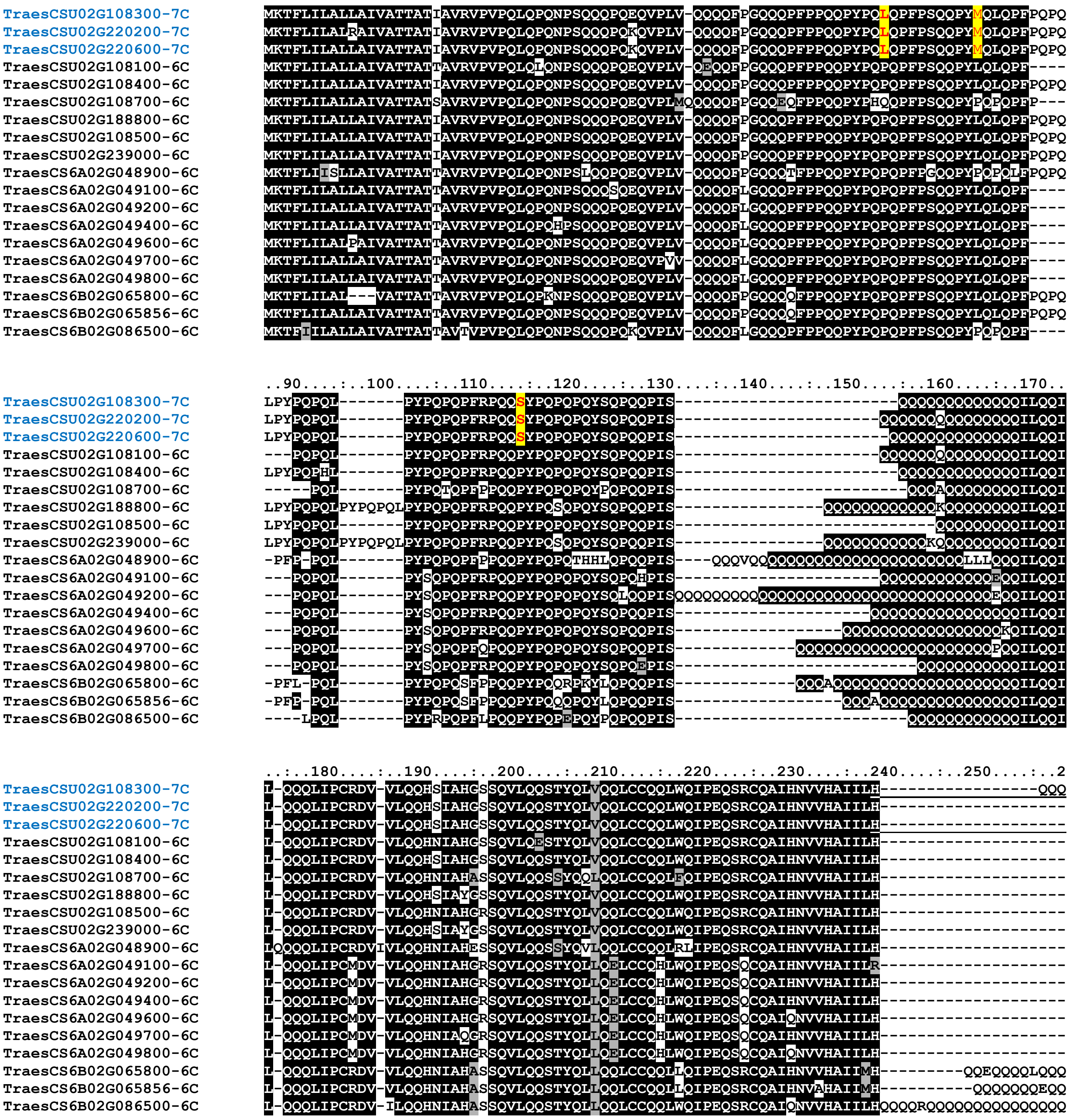

```
                      60....:..270....:..280....:..290....:..300....:..310....:..320....:..330....:..340....
TraesCSU02G108300-7C  QQQQQQQQQQQQQQQQQPLSQVCFQQSQQQYPSGQGSFQPSQQNPQAQGSVQPQQLPQFEEIRNLALETLPAMCNVYIPPYC--TI
TraesCSU02G220200-7C  --QQQQQQQQQQQQQQQPLSQVCFQQSQQQYPSGQGSFQPSQQNPQAQGSVQPQQLPQFEEIRNLALETLPAMCNVYIPPYC--TI
TraesCSU02G220600-7C  --------QQQQQQQQQPLSQVCFQQSQQQYPSGQGSFQPSQQNPQAQGSVQPQQLPQFEEIRNLALETLPAMCNVYIPPYC--TI
TraesCSU02G108100-6C  -QQHHHHQQQQQQQQQQPLSQVSFQQPQQQYPSGQGFFQPSQQNPQAQGSFQPQQLPQFEAIRNLALQTLPAMCNVYIPPYC--TI
TraesCSU02G108400-6C  ----QQQQQQQQQQQQQPLSQVSFQQPQQQYPSGQGSFQPSQQNPQAQGSVQPQQLPQFEEIRNLALETLPAMCNVYIPPYC--TI
TraesCSU02G108700-6C  ---------HHQQQQQQPSSQVSYQQPQEQYPSGQGSFQSSQQNPQAQGSVQPQQLPQFQEIRNLALQTLPAMCNVYIPPYCSTTI
TraesCSU02G188800-6C  -----QQQQQQQQQQQQPLSQVSFQQPQQQYPSGQGSFQPSQQNPQAQGSVQPQQLPQFEEIRNLALETLPAMCNVYIPPYC--TI
TraesCSU02G108500-6C  -------HHQQQQQQQQPLTQVSFQQPQQQYPSGQGSFQPSQQNPQAQGSVQPQQLPQFEEIRNLALETLPAMCNVYIPPYC--TI
TraesCSU02G239000-6C  ----QQQQQQQQQQQKQPLSQVSFQQPQQQYPSGQGSFQPSQQNPQAQGSVQPQQLPQFEEIRNLALETLPAMCNVYIPPYC--TI
TraesCS6A02G048900-6C -QQQQQQQQQQQQGQHQPSSQVSYQQPQQQYPSGQGSFQPSQQNPQAQGFVQPQHLPQLEEISNLALQTLPAMCNVYIPPYCSTTI
TraesCS6A02G049100-6C ---------QQQKQQQQPSSQVSFQQPQQQYPLGQGSFRPSQQNPQAQGSVQPQQLPQFEEIRNLALQTLPAMCNVYIPPYC--TI
TraesCS6A02G049200-6C --------QQQKQQQQQPSSQVSFQQPQQQYPLGQGSFRPSQQNPQAQGSVQPQQLPQFEEIRNLALQTLPAMCNVYIPPYC--TI
TraesCS6A02G049400-6C ---------QQQKPQQQPSSQVSFQQPLQQYPLGQGSFRPSQQNPQARGSVQPQQLPQFEEIRNLALQTLPAMCNVYIPPYC--TI
TraesCS6A02G049600-6C ---------QQQKQQQQPSSQVSFQQPLQQYPLGQGSFRPSQQNPQDQGSVQPQQLPQFEEIRNLALQMLPAMCNVYIPPYC--TI
TraesCS6A02G049700-6C ----QQQKQQQQQQQQQPSSQVSFQQPQQQYPLGQGSFRPSQQNPQAQGSVQPQQLPQFEEIRNLALQTLPAICNVYIPPYC--TI
TraesCS6A02G049800-6C ---------QQQKQQQQPSSQVSFQQPLQQYPLGQGSFRPSQQNPQDQGSVQPQQLPQFEEIRNLALQTLPAMCNVYIPPYC--TI
TraesCS6B02G065800-6C QQQQLQQQQQQQQQQQQPSSQVSFQQPQQQYPSSQVSFQPSQLNPQAQGSVQPQQLPQFAEIRNLALQTLPAMCNVYIPPHCSTTI
TraesCS6B02G065856-6C QQLQQQQQQQLHQQRQQPSSQVSFQQPQQQYPSSQVSFQPSQLNPQAQGSVQPQQLPQFAEIRNLALQTLPAMCNVYIPPHCSTTI
TraesCS6B02G086500-6C QQQQQQQQQQQQQQQQQPSSQVSFQQPQQQYPSGQGSFQPSQQNPQAQSSVQHQQLPQFEEIRNLALQTLPAVCNVYIPPYCSTTI

                      :..350...
TraesCSU02G208300-7C  APVGIFGTN
TraesCSU02G220200-7C  APVGIFGTN
TraesCSU02G220600-7C  APVGIFGTN
TraesCSU02G108100-6C  APFGIFGTN
TraesCSU02G108400-6C  APVGFFGTN
TraesCSU02G108700-6C  APFGIFGTN
TraesCSU02G188800-6C  APVGIFGTN
TraesCSU02G108500-6C  AQVGIFGTN
TraesCSU02G239000-6C  APVGIFGTN
TraesCS6A02G048900-6C APFGIFGTN
TraesCS6A02G049100-6C APFGIFGTN
TraesCS6A02G049200-6C VPFGIFGTN
TraesCS6A02G049400-6C APFGIFGTN
TraesCS6A02G049600-6C APFGIFGTN
TraesCS6A02G049700-6C APFGIFGTN
TraesCS6A02G049800-6C APFGIFGTN
TraesCS6B02G065800-6C APFGIFGTN
TraesCS6B02G065856-6C APFGIFGTN
TraesCS6B02G086500-6C APFGIFGTN
```

Fig. S8. Examples of diagnostic peptides for 7-CYS α-gliadins detected in the proteomics study.

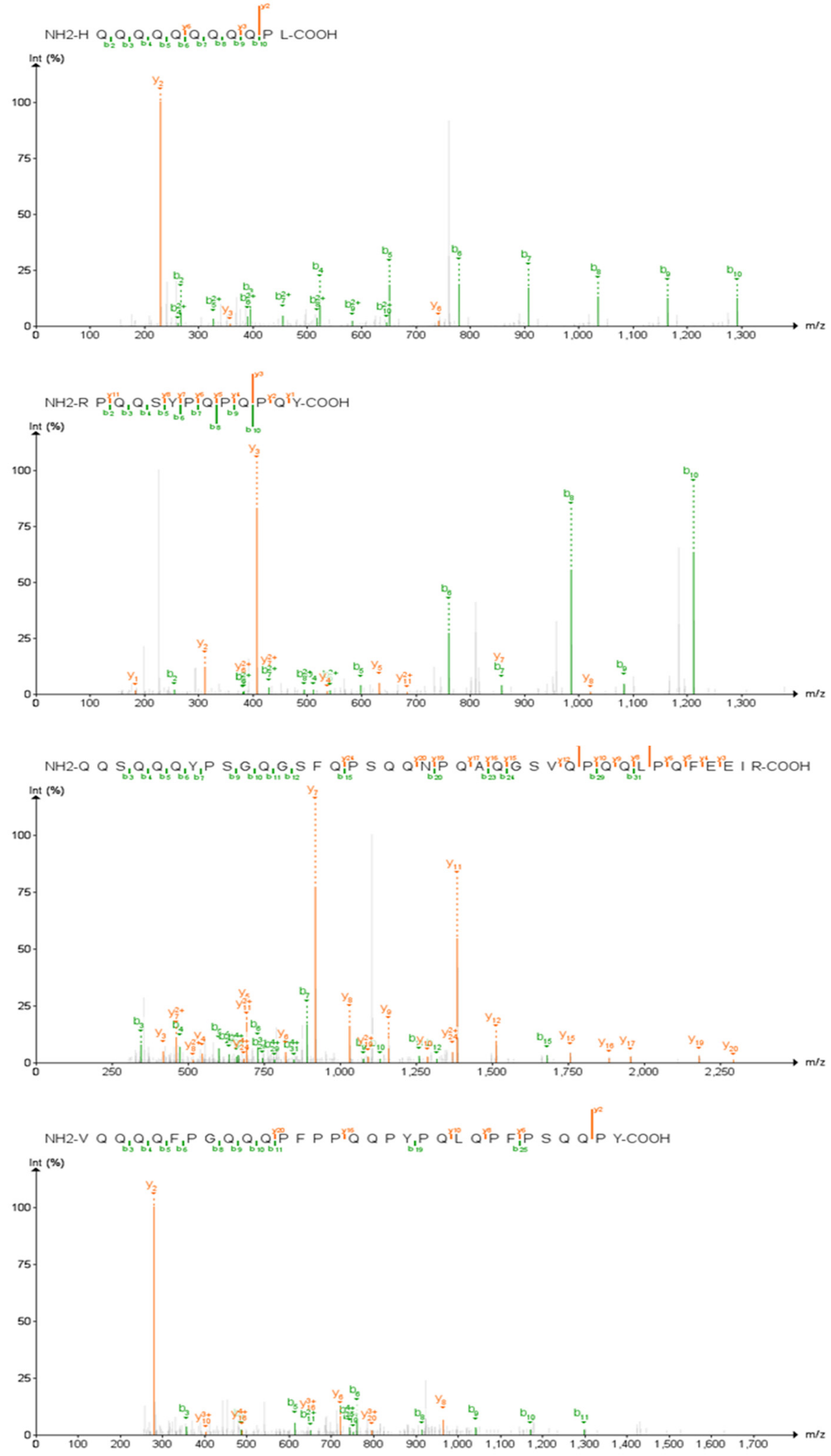

Supplement: Supplementary file 2 [file 122_2025_4882_MOESM2_ESM.pdf]
